# Supplementary material for: BiGAMi: Bi-Objective Genetic Algorithm Fitness Function for Feature Selection on Microbiome Datasets
Source: Methods Protoc. 2022 May 23;5(3):42. doi: 10.3390/mps5030042 (PMC9149982; doi:10.3390/mps5030042)
Supplement: Supplementary file 1 [file mps-05-00042-s001.zip › mps-1679036-supplementary.pdf]

# Supplementary Data

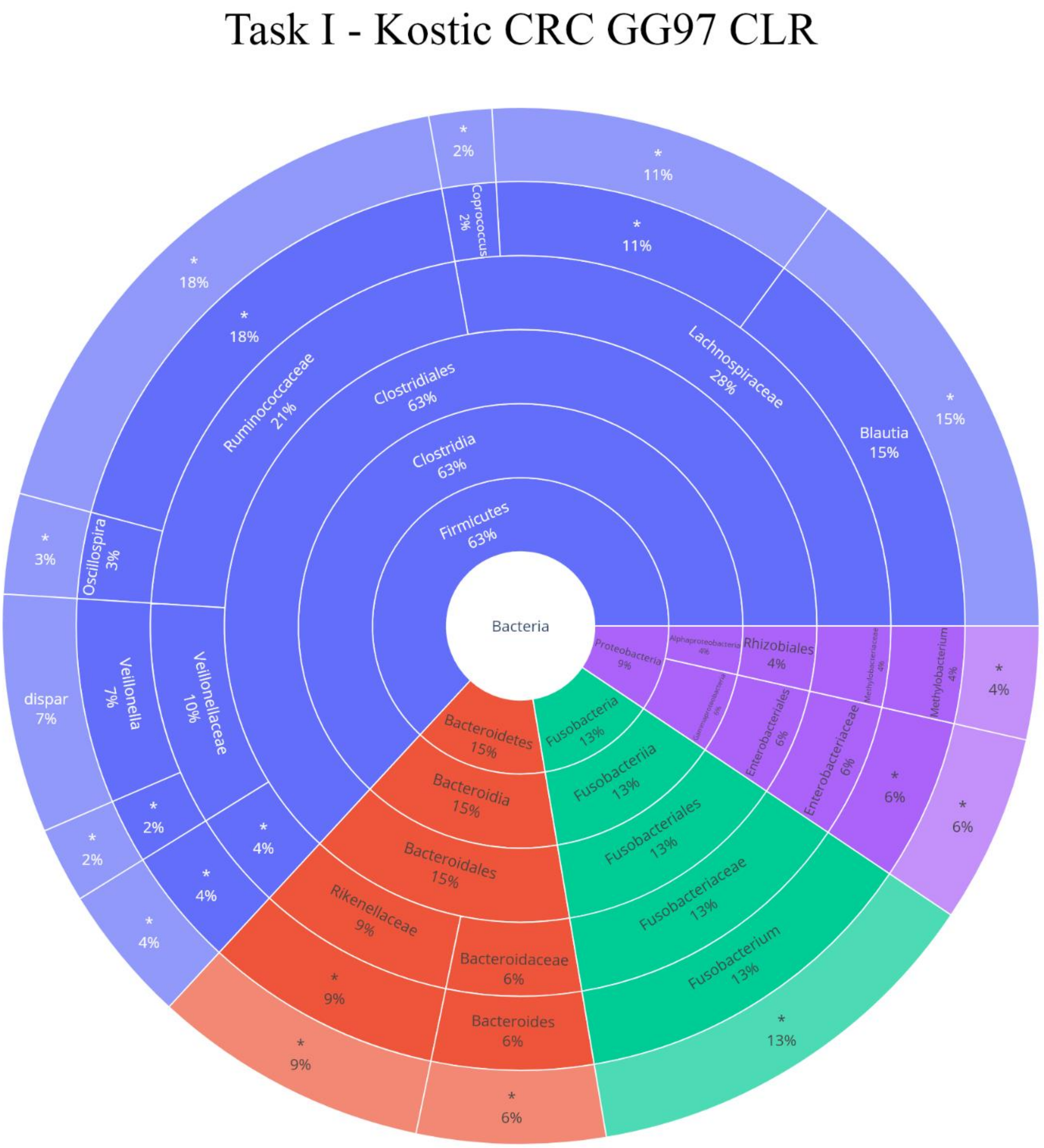


**Figure S1.** Overview of the bacteria selected by the 25 best performing GA individuals for all classification task I: Kostic Colorectal Cancer Healthy/Tumor GG97 CLR.

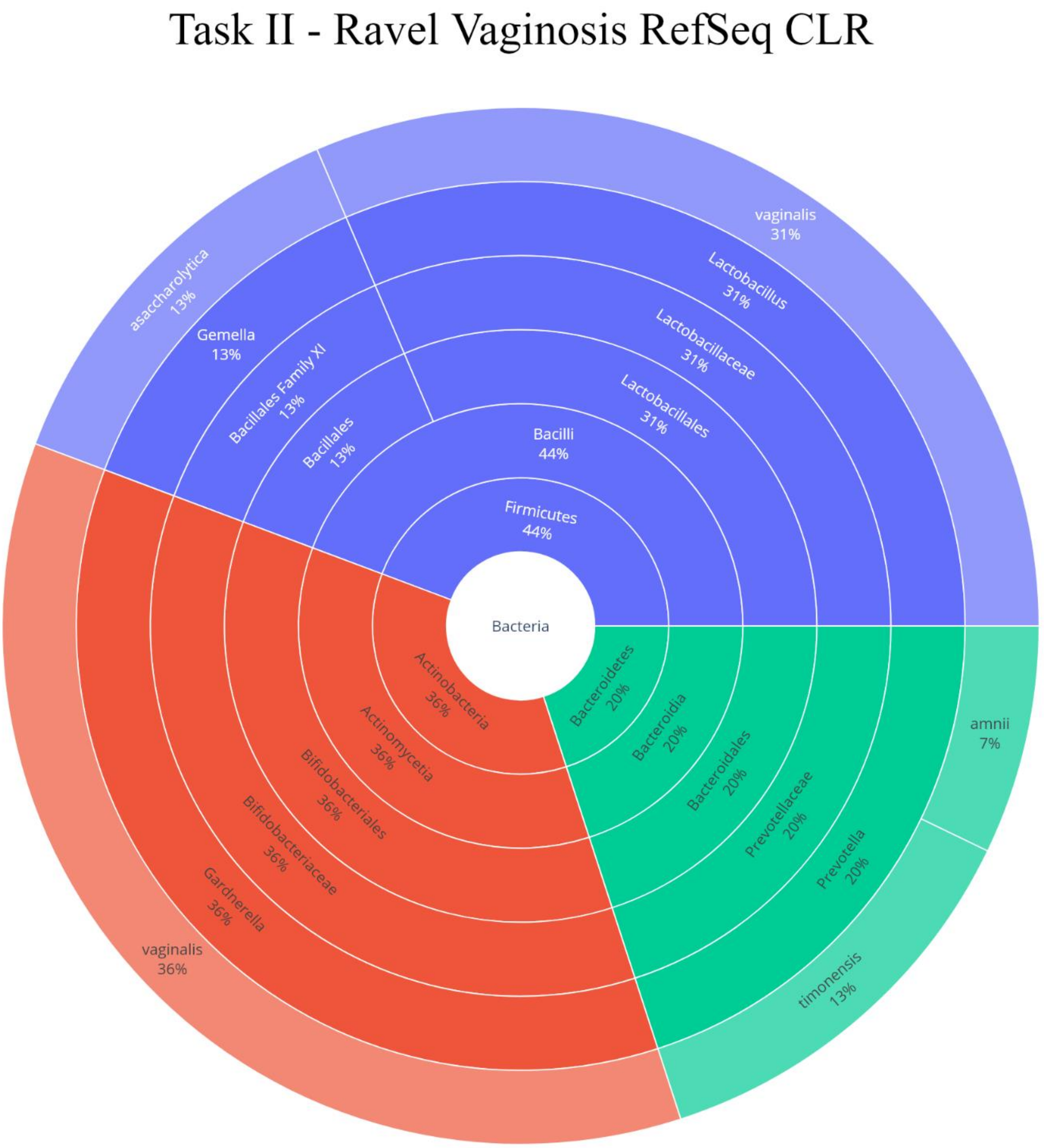


**Figure S2.** Overview of the bacteria selected by the 25 best performing GA individuals for all classification task II: Ravel Vaginal Nugent Category RefSeq CLR.

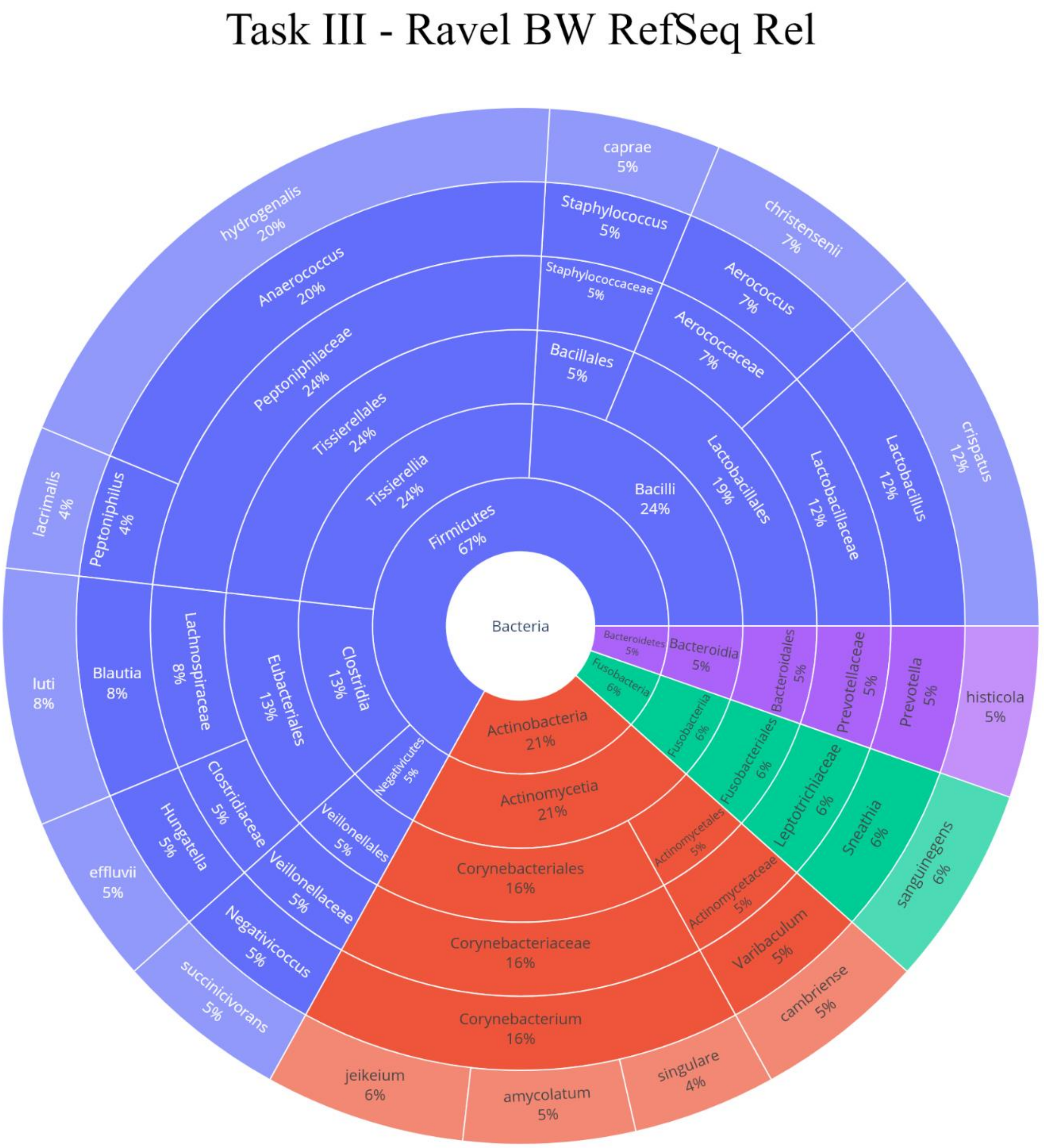


**Figure S3.** Overview of the bacteria selected by the 25 best performing GA individuals for all classification task II: Ravel Vaginal Black/White RefSeq RefSeq CLR.

# Task IV - Qin Cirrhosis RefSeq CLR

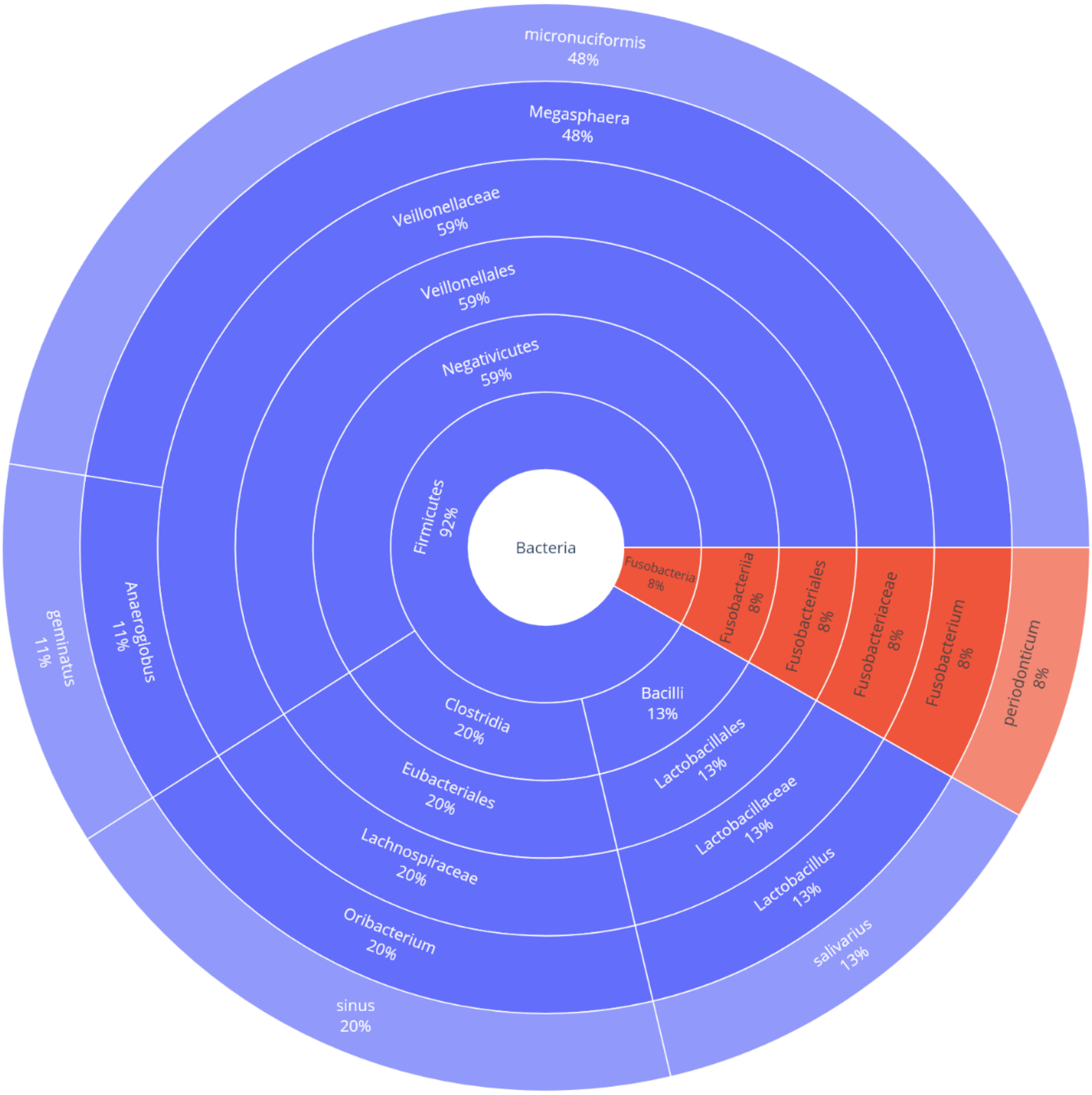


**Figure S4.** Overview of the bacteria selected by the 25 best performing GA individuals for all classification task IV: Qin Cirrhosis RefSeq CLR.
